# Recovery of Regular Daily Physical Activities Prevents Residual Dizziness after Canalith Repositioning Procedures

**DOI:** 10.3390/ijerph19010490

**Published:** 2022-01-03

**Authors:** Salvatore Martellucci, Andrea Stolfa, Andrea Castellucci, Giulio Pagliuca, Veronica Clemenzi, Valentina Terenzi, Pasquale Malara, Giuseppe Attanasio, Francesco Gazia, Andrea Gallo

**Affiliations:** 1 ENT Unit, Ospedale “Santa Maria Goretti”, Azienda USL Latina, 04100 Latina, Italy; dott.martellucci@gmail.com (S.M.); dott.giuliopagliuca@gmail.com (G.P.); veronica.clemenzi@gmail.com (V.C.); terenzivalentina@gmail.com (V.T.); andrea.gallo@uniroma1.it (A.G.); 2 ENT Clinic, Department of Sense Organs, Sapienza University of Rome, 00185 Rome, Italy; 3 ENT Unit, Department of Surgery, Arcispedale Santa Maria Nuova, AUSL—IRCCS, 43123 Reggio Emilia, Italy; andrea.castellucci@ausl.re.it; 4 Department of Odontostomatological e Maxillofacial Sciences, Sapienza University of Rome, 00185 Rome, Italy; 5 Audiology & Vestibology Service, Centromedico Bellinzona, 6500 Bellinzona, Switzerland; pasmalara@gmail.com; 6 Head and Neck Department, Umberto I Policlinic of Rome, 00185 Rome, Italy; giuseppe.attanasio@uniroma1.it; 7 Unit of Otorhinolaryngology, Department of Adult and Development Age Human Pathology “Gaetano Barresi”, University of Messina, 98124 Messina, Italy; ssgazia@gmail.com

**Keywords:** residual dizziness, benign paroxysmal positional vertigo (BPPV), vertigo, canalithiasis, canalith repositioning procedure (CRP)

## Abstract

Objective: Residual dizziness is a disorder of unknown pathophysiology, which may occur after repositioning procedures for benign paroxysmal positional vertigo. This study evaluates the relationship between regular daily physical activity and the development of residual dizziness after treatment for benign paroxysmal positional vertigo. Study Design: Prospective observational cohort study. Setting: Academic university hospital. Methods: Seventy-one patients admitted with benign paroxysmal positional vertigo involving the posterior semicircular canal were managed with Epley’s procedure. Three days after successful treatment, the patients underwent a telephone interview to investigate vertigo relapse. If the patients no longer complained of vertigo, they were asked about symptoms consistent with residual dizziness. Subsequently, they were asked about the recovery of physical activities they regularly performed prior to the onset of vertigo. Results: Sixty-nine patients (age: 57.79 ± 15.05) were enrolled: five (7.24%) reported vertigo relapse whereas twenty-one of sixty-four non-relapsed patients (32.81%) reported residual dizziness. A significant difference in the incidence of residual dizziness was observed considering the patients’ age (*p* = 0.0003). Of the non-relapsed patients, 46 (71.88%) recovered their regular dynamic daily activities after treatment and 9 (19.57%) reported residual dizziness, while 12 of the 18 patients (66.67%) who did not resume daily activity reported residual symptoms (*p* = 0.0003). A logistic regression analysis showed a significant association between daily activity resumption and lack of residual dizziness (OR: 14.01, 95% CI limits 3.14–62.47; *p* = 0.001). Conclusions: Regardless of age, the resumption of regular daily physical activities is associated with a lack of residual dizziness.

## 1. Introduction

Benign paroxysmal positional vertigo (BPPV) is the most common vestibular complaint, consisting of short-lasting vertigo spells triggered by head position changes. BPPV affects both utricular macula and one or more semicircular canals as the most accredited pathophysiology is the displacement of otoconial matter from the utricle to the involved canal. Free-floating otoconia modify endolymphatic flows and cupular responses during head movements, resulting in positional vertigo and nystagmus [1,2].

BPPV can be classified, in clinical practice, according to the affected semicircular canal (SC) and the involved arm, as follows [1,2]:

- Posterior Semicircular Canal (PSC) BPPV: Geotropic and apogeotropic variants
- Horizontal Semicircular Canal (HSC) BPPV: Geotropic and apogeotropic variants
- Anterior Semicircular Canal (ASC) BPPV
- Multicanal BPPV

The involvement of a single SC represents the most frequent condition, although BPPV might simultaneously affect more SCs on one or both sides. PSC BPPV is the most frequent variant (80–90%), whereas cases involving the HSC and ASC account for 10–20% and 3% of all BPPVs, respectively [3]. In addition, canalith jam (CJ) is an uncommon variant of vestibular lithiasis [4,5,6].

The diagnosis of BPPV concerning the affected ear and the involved canal and arm is commonly made by performing positioning tests, also known as diagnostic maneuvers. The tests work by gravity and inertial/centrifugal forces, which bend the affected SC cupula and move the free-floating debris [1,2,3,7]. Several maneuvers have been proposed to properly diagnose each BPPV variant by moving the patient’s head along the plane of the examined SC [1,2,3]. The “minimum stimulus strategy” represents a nystagmus-based approach aimed at minimizing the patient’s discomfort, reducing the amount of diagnostic and therapeutic maneuvers required for BPPV diagnosis and treatment [8,9].

BPPV can be effectively managed by repositioning maneuvers, namely non-invasive procedures meant to move back displaced debris towards the utriculus. The canalith repositioning procedure (CRP) proposed by Epley is the most commonly used technique for the treatment of BPPV involving the PSC, which represents the most common subtype of BPPV [1,3].

Following successful repositioning maneuvers for BPPV, patients often experience light-headedness, short-lasting unsteadiness, or a persistent non-positional imbalance of variable duration. These residual symptoms, also called residual dizziness (RD), more frequently occur in older patients and in subjects with anxiety-related disorders [10,11,12,13]. Several hypotheses have been proposed to explain the pathomechanism underlying residual symptoms after successful repositioning procedures. Possible explanations include either the persistence within the canal lumen of a too limited number of residual debris to provoke detectable positional nystagmus, the persistence of utricular dysfunction accompanying BPPV, and the occurrence of long-lasting central adaptation mechanisms [14,15,16,17,18,19,20,21]. In previous research, the overall prevalence of RD ranges between 36.6% and 61% [10], and both vestibular rehabilitation and various drugs have been proposed to manage this condition [22,23,24].

This study evaluates the relationship between the resumption of regular daily physical and the development of RD after the successful treatment of PSC-BPPV.

## 2. Materials and Methods

In this prospective observational trial, we enrolled 76 patients (30 male and 46 female) admitted to the emergency room for vertigo who were diagnosed with PSC-BPPV.

Patients affected by other BPPV forms, multiple semicircular canals involvement or with a history consistent with previous vestibular disorders other than BPPV were excluded, as were patients who missed the scheduled follow-up visits. Subjects with simultaneous temporary physical impediments associated with BPPV (for example, traumatic pathologies reducing the ability to move) were also excluded.

All the patients received a bedside neurotological evaluation, including an examination of ocular alignment, saccades, smooth pursuit, and gait. Both spontaneous and gaze-evoked nystagmus with and without fixation were checked using infrared video-Frenzel goggles. According to our nystagmus-based approach, the patients underwent diagnostic positioning tests for BPPV according to the minimum stimulus strategy [25,26]. PSC-BPPV was diagnosed if the Dix–Hallpike test evoked typical paroxysmal nystagmus (up-beating and torsional nystagmus with the upper pole of the eyes beating toward the undermost ear, lasting < 1 min).

If the positional tests were consistent with PSC-BPPV, CRP, as described by Epley [3], was immediately performed. No drug was prescribed before or after physical therapy. A Dix–Hallpike maneuver was repeated about 5 min after the treatment and CRP was defined as “successful” if positional nystagmus was no longer detectable and the patient did not complain of vertigo at the control diagnostic test. Otherwise, the first CRP was defined as “ineffective” and repeated up to three times, checking the treatment result following each maneuver. In cases of ineffective CRP, the patients were re-evaluated after three days with the same protocol. A maximum of two sessions (six CRP) per patient was carried out. Patients were excluded from the study if more than two sessions were required or a canal switch occurred.

Three days after successful CRP, each patient underwent a telephone interview consisting of three dichotomous questions, to which the only possible answers were “YES” or “NO”. The first question, aimed to identify the persistence of dizziness during rotational and flexion-extension head movements, was: “Do you still feel dizzy when moving your head, lying down, turning in bed and getting up from supine?”. In case the answer was “YES” (persistence of positional symptoms), the interview concluded, the patient was classified as “relapses” and was invited to return to our clinic for a further check-up. Conversely, two more questions were asked if the answer was “NO” (receding of positional symptoms). The second question, aimed to verify the onset of RD, was: “Do you currently feel unsteady as you were not before the onset of BPPV?”. Patients who answered “YES” were considered to have RD. Finally, the patients were asked about the resumption of their regular dynamic daily activities (RDDA) after physical therapy through the third question: “After the last successful maneuver, have you resumed the daily physical activities you were performing before the onset of BPPV?”. RDDA were defined as any physical activity that patients conducted before the onset of BPPV, considering differences related to age, habits, and performance status: playing sports, swimming, jogging, cycling, walking, climbing stairs, and performing housework.

**Figure 1:**
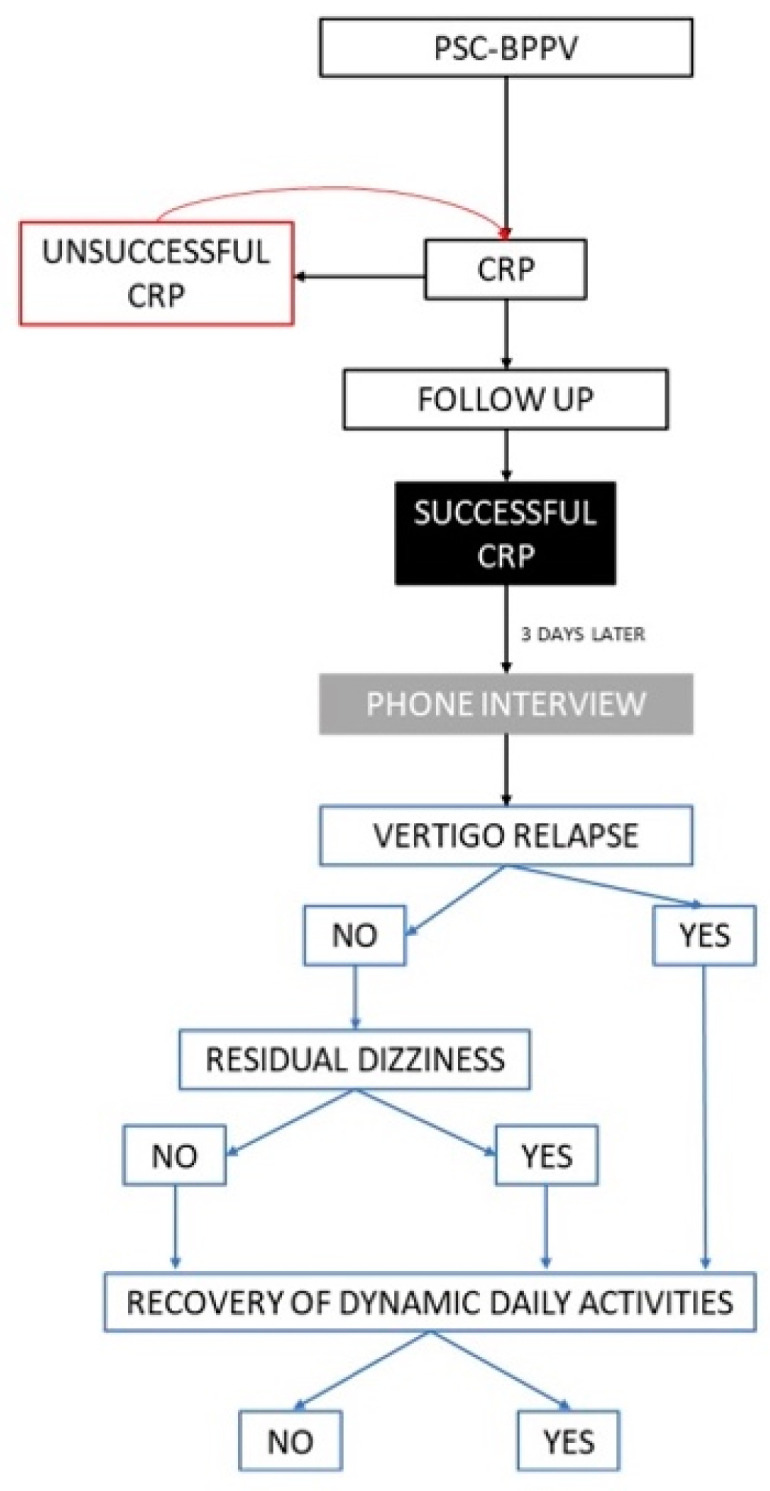
Study design. BPPV, benign paroxysmal positional vertigo; CRP, canalith repositioning procedure; PSC-BPPV, posterior semicircular canal BPPV.

The study design is presented in Figure 1.

### Statistical Analysis

The continuously distributed variables were reported as mean +/− SD, after checking for normal distribution, and compared by the Student’s *t*-test. The frequencies and percentages were calculated for the categorical variables, and the chi-square test was used for the comparisons.

A multivariable logistic regression model with backward stepwise variable selection was constructed to assess which factors were independently associated with the lack of RD among those highlighted in the univariate setting (sex, age, affected side, number of CRP performed, resumption of daily activities). Any *p*-value < 0.05 was considered statistically significant.

## 3. Results

Among the overall cohort of patients enrolled in the trial (n. 71), seven subjects were later excluded since five (7.04%) failed to attend the follow-up visits, while two (2.81%) required more than six CRPs.

Sixty-nine subjects were eventually included in the statistical analysis: 30 males (43.47%, mean age: 58.8 ± 13.80) and 39 females (53.13%, mean age: 57.02 ± 16.08). The affected ear was the right ear in 44 cases (63.76%). In total 39 patients (56.52%) required a single CRP to recover from PSC-BPPV, 14 (20.28%) received two CRPs, 11 (15.94%) required three CRPs, while 5 subjects received up to 6 CRPs (7.24%). The demographic data are summarized in Table 1.

At the telephone interview, five patients (7.24%) reported relapse of vertigo and among them, only three reported a prompt recovery of RDDA after the treatment. No differences were found in relapse occurrence according to age, sex, affected side, and number of CRPs.

**Figure 2:**
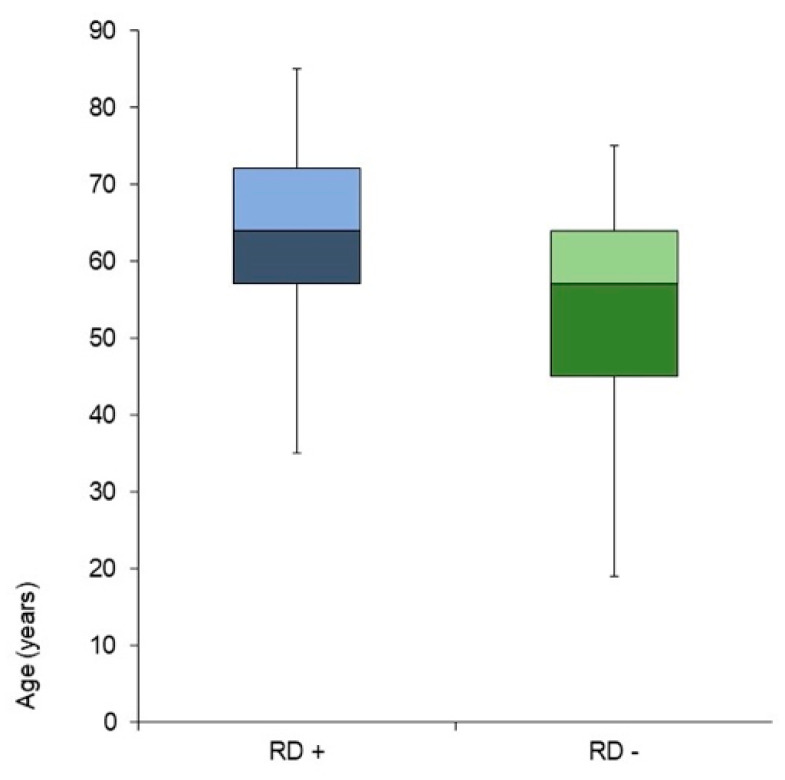
Residual dizziness reporting according to age. RD+, patients reporting residual dizziness; RD−, patients free from residual dizziness.

**Figure 3:**
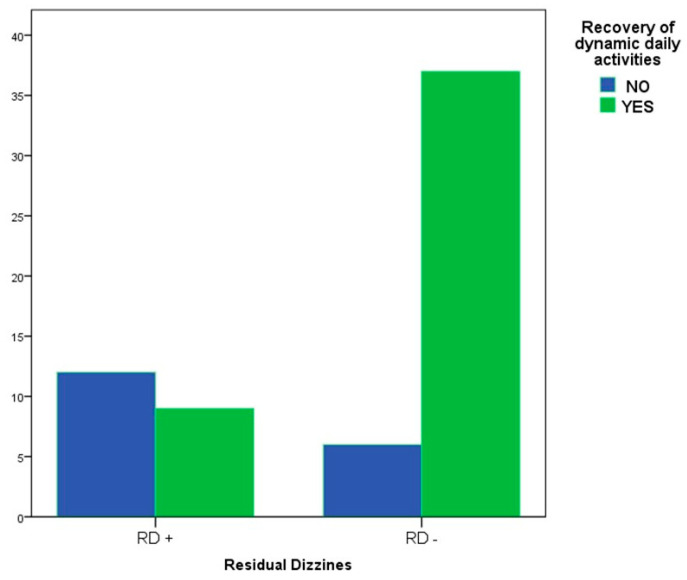
Correlation between residual dizziness and recovery of dynamic daily activities. RD+, patients reporting residual dizziness; RD−, patients free from residual dizziness.

Among the remaining 64 patients, 21 (32.81%) complained of RD. The statistical analysis did not show differences in RD incidence according to sex, affected side, or number of CRP. By contrast, a significant difference in the incidence of RD was found considering age: the patients who experienced RD were significantly older than those without RD (64.24 ± 12.67 vs. 53.98 ± 14.82, *p* < *0*.005) (Figure 2). Among the non-relapsed patients (n. 64), 46 (71.88%) reported a complete resumption of RDDA after treatment, and 9 (19.57%) complained of RD. Conversely, among the remaining 18 patients who denied having resumed their daily activities, 12 subjects (66.67%) developed RD (*p* = 0.0003) **(**Figure 3).

The multivariable logistic regression model showed that patients who promptly recovered RDDA were 14 times less likely to experience RD, irrespective of sex, age, and number of CRP (OR: 14.01, 95% CI 3.14–62.47; *p* = 0.001).

## 4. Discussion

RD is an ambiguous disorder occurring after the successful treatment of BPPV, once positional vertigo has receded. Patients often struggle to report RD, since the disorder includes a broad spectrum of symptoms, such as a vague sense of dizziness, imbalance, light-headedness, or other balance disorders without vertigo [12].

RD is considered a complex vestibular disorder and several hypotheses have been proposed to explain its pathophysiology [14,15,16,17,18,19,20,21]. Although theoretically valid, none of them has been supported by definitive data, nor do they exclude other causal factors that could play a role in the genesis of RD acting either independently or synergistically.

Some authors assume that residual symptoms could represent a subclinical variant of BPPV due to the persistence of debris in the semicircular canal lumen. The residual otoconial matter, they argue, results in mild dizziness, although it does not provoke positional nystagmus or vertigo [18,27].

Other hypotheses focus on the role of a possible dysfunction in the utricular macula, in association with or due to BPPV. This condition could be defined as “maculo-canalopathy”; therefore, physical therapy can solve the canal dysfunction via debris removal without restoring macular function [1,2,3]. In support of this theory, Von Brevern et al. assessed the otolith-ocular reflex (OOR), suggesting that idiopathic BPPV is associated with utricular dysfunction and assumed that this condition could result in long-lasting imbalance after the resolution of canalolithiasis [28]. This supposition was later supported by other studies [17,29]. Furthermore, macular dysfunction due to traumatic or degenerative otoconial loss have recently been proposed as causative factors for RD [20,21].

Faralli et al. suggested that the genesis of RD could be related to the inability of the vestibular system to readjust to a new functional status quickly [16]. According to this theory, otoconial debris within the semicircular canals alter the tonic discharge of the affected labyrinth during BPPV, thereby inducing a central adaptation to rebalance the activity of vestibular nuclei, reducing peripheral asymmetry. The sudden resolution of BPPV by physical therapy alters the “new equilibrium” achieved. Therefore, central adaptation cannot promptly restore the pre-existing condition and produces subtle symptoms consistent with RD. The delayed central adaptation is associated with many factors, such as BPPV duration [10,11], age [10,11,12,13,14,15,16], and the subject’s emotional state [12,13,14]. Furthermore, it should be considered that aging significantly degrades sensory systems, worsening central processes measuring balance and body perception, therefore predisposing to dizziness and disequilibrium [30]. It is well known how advancing age could increase the incidence of RD, representing an important variable to be considered [13,15]. These data are in accordance with the results of this study and with data already collected in our previous investigation [12], in which we described how age influenced the occurrence of imbalance after successful CRP. Since many factors can affect the risk of RD development, this trial included only patients with canalolithiasis affecting the posterior semicircular canal to eliminate confounding aspects related to therapeutic procedures other than CRP and misdiagnosis (i.e., central vertigo or cupulopathies mimicking BPPV). Furthermore, previous research about this topic showed that diagnostic delay is linked with a higher probability of developing RD. Therefore, only patients with recent-onset BPPV were enrolled in this trial, in order to limit the confounding factors and make the analyzed cohort more homogeneous. The presence of anxiety-related disorders, a relevant aspect in the development of residual symptoms after the resolution of BPPV, was not investigated in this earlier study.

This study mainly investigated the association between RDDA and RD. We considered the resumption of RDDA as a purely qualitative and subjective parameter, which was assessed by asking patients whether they resumed the motor activities they usually performed before the first vertigo spell after successful CRP. Each subject follows a daily routine involving repeated motor activities, regardless of age and habits. This routine varies from case to case: naturally, the RDDA of a young and fitness-focused patient will be different from that of an elderly subject with a sedentary lifestyle. However, considering our population’s differences according to age and habits, attempting to quantify each patient’s physical activity before and after BPPV would have been complicated and probably not very accurate. A solid association between RDDA resumption and the absence of RD, regardless of the subjects’ age, was found. The data obtained from our analysis can be interpreted in two ways: the RDDA may prevent RD but, conversely, RD could prevent the resumption of the RDDA. The first hypothesis aligns with the different proposed pathogenetic theories underlying RD.

The results obtained from our analysis are in line with the different proposed pathogenetic theories underlying RD. For example, after successful repositioning maneuvers, the head movements associated with daily motor activities might be able to disperse the residual otoconial fragments in the canal lumen, preventing further perturbations on the cupula.

Although RD is mostly a self-limiting condition, patients can sometimes develop enough discomfort and disabling symptoms to require physical treatment and vestibular rehabilitation, in combination with medications if needed [31].

Drugs play an unclear role in the management of patients suffering from RD. Several medications have been proposed to approach RD, but nothing has yet been proven to provide relief for this disorder compared to placebo [13]. Albeit with conflicting results, the most commonly used molecule to approach RD is betahistine [22,24,31]. According to a randomized controlled trial, dimenhydrinate is a vestibular suppressant that could help prevent RD. However, it presents some side effects, limiting its use by selected patients for a limited number of days [32]. A prospective multicentric study reported the beneficial effect of supplementation with a polyphenol compound [23].

Vestibular rehabilitation has been developed to treat patients with chronic vestibular symptoms through a tailored exercise-based program. Vestibular rehabilitation, thanks to the development of vestibular habituation, adaptation, and substitution to enhance gaze and postural stability, is proven to improve daily functional performance [31,33].

Rodrigues et al. demonstrated that vestibular exercises in association with CRP could increase the benefit of treatments for patients with BPPV, resulting a reduction in residual symptoms and a decreasing recurrence rate [34]. Therefore, considering RD as the effect of either macular imbalance or delayed central compensation, RDDA would ideally act similarly to vestibular rehabilitation, readjusting the affected utricular function, although without the proper structure and goals. Assuming that the daily activities that are usually performed consist of systematic repetitions of space-oriented movements, it could be hypothesized that the resumption of RDDA could act centrally, rebalancing the peripheral asymmetry that occurs while otoconia are free to move within the semicircular canal. However, considering the hypothesis of permanent utricular dysfunction, the role of RDDA in preventing RD appears justified, since it might enhance neuroplasticity phenomena, making the vestibular system more receptive to natural compensatory mechanisms [34].

Nevertheless, some authors have associated the resumption of physical activity after physical therapy with BPPV relapse. For this reason, postural restrictions in the first days following physical therapy have been proposed, although this practice appears to be unjustified based on current evidence [35,36]. In our series, no differences were found in BPPV relapse subsequent to RDDA, according to previous studies.

The present study features some limitations. Firstly, it involved only patients recruited in the emergency room, with recent-onset BPPV. Since the occurrence of RD is positively related with the duration of BPPV [10,11], we could not establish whether RDDA would have played the same role in a cohort of patients with long-lasting BPPV. Furthermore, the vestibular function was not investigated instrumentally after physical therapy nor, at the follow-up evaluation. Thus, we could not analyze the effects of RDDA in case of hypothetical utricular damage.

## 5. Conclusions

The prompt resumption of RDDA is associated with a lack of RD after physical therapy in patients admitted to the emergency room with BPPV, regardless of age and without increasing the risk of relapses.

## Figures and Tables

**Table 1:** Demographic Data. DDA: Recovery of dynamic daily activities; SD: standard deviation; R: Right; L: Left; R.VERT: Relapse of vertigo; CRP: Canalith repositioning procedure; RD+: Residual dizziness development.

DDA	Subjects		Age (Mean ± SD)		Side		R. VERT	Number of CRP				RD+	RD−
					R	L		1	2	3	>4		
Yes	Male	22	58.81	±12.90	16	6	-	11	8	2	1	4	18
	Female	27	56.70	±18.10	16	11	3	19	3	3	2	5	19
No	Male	8	58.75	±17.04	5	3	-	4	1	3	-	5	3
	Female	12	57.50	±10.87	7	5	2	5	2	3	2	7	3
Tot.		69	57.79	±15.05	44	25	5	39	14	11	5	21	43

## Data Availability

The data presented in this study are available on request from the corresponding author. The data are not publicly available due to the privacy.
